# Drought-Responsive NAC Transcription Factor *RcNAC72* Is Recognized by *RcABF4*, Interacts with *RcDREB2A* to Enhance Drought Tolerance in Arabidopsis

**DOI:** 10.3390/ijms23031755

**Published:** 2022-02-03

**Authors:** Xin Jia, Zhen Zeng, Yingmin Lyu, Shiwei Zhao

**Affiliations:** 1 Beijing Key Laboratory of Ornamental Germplasm Innovation and Molecular Breeding, China National Engineering Research Center for Floriculture, College of Landscape Architecture, Beijing Forestry University, Beijing 100083, China; jxin95@bjfu.edu.cn (X.J.); zengzhen@bjfu.edu.cn (Z.Z.); 2 Beijing Key Laboratory of Greening Plant Breeding, Beijing Institute of Landscape Architecture, Beijing 100201, China

**Keywords:** NAC72, ABF4, DREB2A, regulation, rose, drought stress

## Abstract

*RcNAC72*, a key transcription factor that may respond to drought stress in *Rosa chinensis* ‘Old Blush’, was selected in our previous study. In the present study, we found that *RcNAC72* is localized in the nucleus and is a transcriptional activator. *RcNAC72* expression could be significantly induced by drought, low temperature, salt as well as abscisic acid (ABA) treatment. Analysis of the promoter revealed that multiple abiotic stress and hormone response elements were located in the promoter region. The promoter could respond to drought, low temperature, salt and ABA treatments to activate GUS gene expression. Overexpressing *RcNAC72* in Arabidopsis thaliana enhanced sensitivity to ABA and tolerance to drought stress. Silencing of *RcNAC72* by virus-induced gene silencing (VIGS) in rose leaves significantly reduced leaf water loss tolerance and leaf extension capacity. Physical interaction of *RcNAC72* with *RcDREB2A* was shown by means of the yeast two-hybrid (Y2H) and bimolecular fluorescence complementation (BiFC) assays. *RcABF4* was demonstrated to be able to bind to the promoter of *RcNAC72* by means of the yeast one-hybrid (Y1H) assay. These results provide new insights into the regulatory network of *RcNAC72* response to drought stress in roses.

## 1. Introduction

Drought is one of the most important abiotic stresses, and drought stress is increasing with climate warming [1,2]. Drought stress inhibits plant growth, development and yield [3]. Plants have evolved a range of regulatory mechanisms to adapt to drought. These responses are a complex regulatory network. Transcription factors, as regulatory proteins, can specifically recognize cis-acting elements and play a crucial role as molecular switches that regulate downstream genes expression [4]. Studies have shown that different types of TFs exhibit vital roles in plant response to drought stress [5,6,7,8]. In order to understand the regulatory network of plant response to drought stress, one can start with the study of transcription factors.

NAM, ATAF and CUC (NAC) constitute one of the largest families of plant-specific transcription factors. *NACs* contain a conserved *N*-terminal associated with DNA binding and a highly distinct *C*-terminal region involved in transcriptional activation [9]. *NACs* have been elucidated to be involved in plant abiotic stress regulation and may be a promising candidate for improving stress tolerance in plants [10]. In apple, the *MdSND1* gene was induced by salt, mannitol and ABA, and overexpressed apple plants have a stronger ability to resist osmotic stress [11]. The *MdNAC047* gene was isolated and functionally characterized as involved in ethylene regulation of salt tolerance [11]. Overexpression of *ANAC016* [12], *ANAC019*, *ANAC055*, *ANAC072* [13] and *ANAC096* [14] improved the abiotic stress ability of transgenic Arabidopsis plants. Functional studies on potato *StNAC053* [15], durum wheat *TtNAC2A* [16], *CaNAC46* [17], rice *ONAC022* [8], *ONAC066* [18] and *OoNAC72* [19] genes showed that all these genes could improve the tolerance of transgenic plants to drought stress. Similarly, wheat *TaNAC29* [20] and *TaNAC48* [21] both improved drought resistance in transgenic Arabidopsis, these genes could be candidates for enhancing drought resistance in wheat. *SlNAC11* [22], *SlJUB1* [3] and *SlNAC6* [23] were shown to be positive regulators of drought tolerance in tomato by RNAi silencing and transgenic. Overexpression of kiwi *AvNAC030* in Arabidopsis has higher osmoregulatory capacity and antioxidant capacity [24]. *NACs* associated with water loss stress and ethylene-induced transcription were screened in cDNA microarray analyses of water loss stress in cut rose [25]. Studies on *RhNAC2* [25] and *RhNAC3* [26] found that both increased tolerance to water loss stress by regulating the expression of downstream genes. Similarly, the *RhNAC31* gene acted as a positive transcriptional regulator in response to multiple abiotic stresses [27]. Drought tolerance was enhanced by DgNAC1 overexpression in chrysanthemum [28].

The transcriptional regulatory response of plants to drought stress was divided into ABA-dependent and ABA-independent signal transduction pathways [29]. It was found that ABA plays essential roles in water deficit response [4]. Many NAC transcription factors such as rice *OsNAC3* [30], maize *ZmSNAC13* [31] and *ZmNAC33* [32] have been reported to be induced and up-regulated by exogenous ABA and be involved in an ABA-dependent signaling pathway in response to abiotic stresses. In Arabidopsis, *ANAC016* directly bound to the abscisic acid-responsive element binding protein 1 (ARBE1) promoter and repressed *AREB1* expression [12]. *ANAC072* responded to ABA expression and interacted with ABA response element binding factor 3 (*ABF3*) [33]. *ANAC096* directly interacted with *AtABF2* and *AtABF4* to help plants survive under dehydration [14]. In wheat, *TaABRE2* bound to the ABRE cis-acting element on the *TaNAC48* promoter, indicating that *TaNAC48* was involved in the ABA signaling pathway in response to drought stress in wheat [21]. *PwNAC11* could interact with *ABF3* to improve drought tolerance in transgenic Arabidopsis [34]. In addition, studies have elaborated that NAC TFs have regulatory relationships with DREB TFs. *AtJUB1* could directly activate the expression of *AtDREB2A* and the tomato *SlJUB1* could bind to the promoter of *SlDREB1* and *SlDREB2*, involved in regulating drought response [3]. Lily *LlNAC2* can interoperate with *LlDREB1* in response to low temperature stress [35]. *PwNAC11* can interact with *PwDREB2A* to enhance drought tolerance [34].

*Rosa chinensis* ‘Old Blush’ is an ancient Chinese species involved in modern rose breeding, and can be used as a model plant for studying the response of roses to drought stress. In our previous study, *RcNAC72* was found to be a key transcription factor gene in response to drought stress in roses [36]. We conducted further studies on *RcNAC72* in this report. *RcNAC72* was induced by drought, low temperature, salt and ABA. Similarly, the promoter of *RcNAC72* was also induced by drought, low temperature, salt and ABA. *RcABF4* recognized the ABRE cis-acting element in the *RcNAC72* promoter region, and *RcNAC72* interacted with *RcDREB2A*. Furthermore, silencing of *RcNAC72* by VIGS in rose leaves significantly reduced leaf water-loss tolerance. Overexpression of *RcNAC72* transgenic Arabidopsis showed greater tolerance to drought stress and enhanced sensitivity to ABA. These above results illustrate that *RcNAC72* is involved in the ABA signal transduction pathway in response to drought stress. Our study provides new insights into the regulatory mechanisms of *NACs* in response to drought stress in roses.

## 2. Results

### 2.1. Bioinformatics Analysis of RcNAC72

*RcNAC72* contained an open reading frame (ORF) of 1059 bp. It is a protein comprising 352 amino acids with an isoelectric point of 8.38 and a theoretical molecular weight of 39,746.04 kD. Protein multiple sequence alignment analysis showed that *RcNAC72* protein had a conserved NAC domain in the *N*-terminal region which can be divided into five subdomains, A to E (Figure S1A). Phylogenetic analysis indicated that *RcNAC72* was clustered closely to *Fragaria vesca FvNAC72*, and has 91% identity to *ANAC72* (Figure S1B). Similarly, for the BLAST sequence of *RcNAC72* using The Arabidopsis Information Resource (TAIR), the results show that *ANAC72* produced significant alignments.

**Figure 1:**
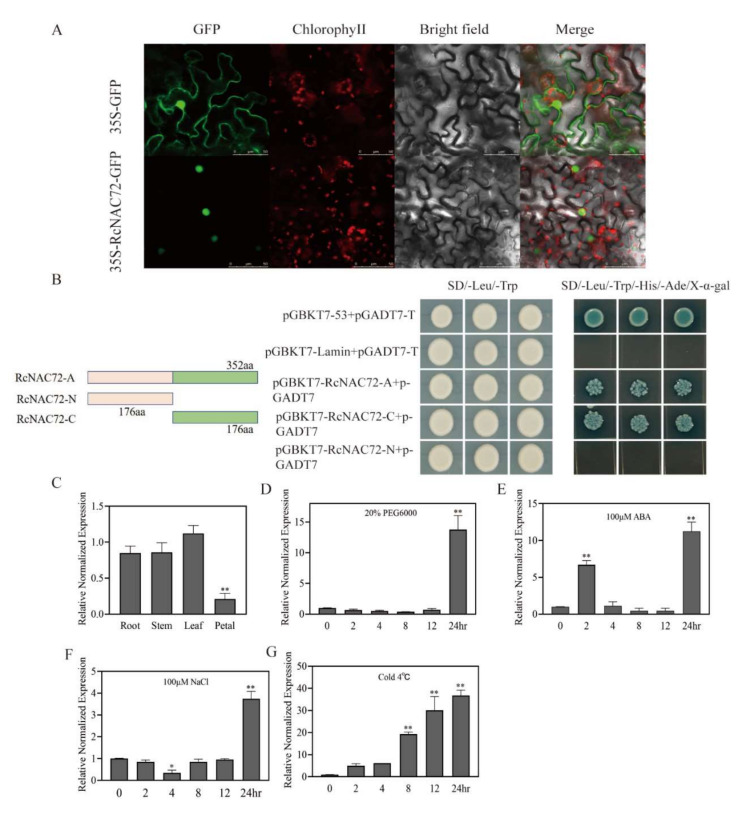
Subcellular localization, transcriptional activation, and stress induction of *RcNAC72* in roses. (**A**) Subcellular localization of *RcNAC72*. (**B**) Transcriptional activation of *RcNAC72*. (**C**–**G**) Analysis of the tissue specificity and expression pattern under abiotic stress treatment of *RcNAC72*. Three biological replications were performed. Pink color represents the 176 amino acids at the N-terminal. Green color represents the 176 amino acids at the C-terminal. The bars show the SD. Asterisks indicate a significant difference ** *p* < 0.01 and * *p* < 0.5 compared with the corresponding controls.

In the tobacco transiently transformed with pBI121-35S-GFP and pBI121-35S-*RcNAC72*-GFP, GFP was expressed ubiquitously. In the tobacco transiently transformed with pBI121-35S-GFP, the green fluorescent signal was distributed in the cell membrane and nucleus, but the pBI121-35S-*RcNAC72*-GFP fusion protein fluorescence signal was only detected in the nucleus (Figure 1A), demonstrating that *RcNAC72* was a nuclear protein.

The yeast two-hybrid assay was conducted to detect whether *RcNAC72* had transcriptional activating activity. The full-length *RcNAC72* gene (*RcNAC72*-A), *N*-terminal (*RcNAC72*-N) and *C*-terminal (*RcNAC72*-C) were inserted into pGBKT7 to form recombinant plasmids. The pGADT7 plasmid and recombinant plasmids co-transformed into yeast cells that could be cultured well on the selection medium SD/-Leu/-Trp, indicating that they were transferred into yeast cells (Figure 1D). Co-transformed yeast cells containing pGBKT7-*RcNAC72*-A and pGBKT7-*RcNAC72*-C plasmids could grow well on the selection medium SD/-Trp/-His/-Ade-x-α-gal and appeared blue, suggesting that *RcNAC72* is a transcriptional activator, and its transactivation domain is located in the *C*-terminal region (Figure 1B). The co-transformations of yeast cells with pGBKT7 and pGADT7-T were positive controls, and those with pGBKT7-Lamin and pGADT7-T were negative controls.

### 2.2. RcNAC72 Tissue Specificity and Expression Analysis under Abiotic Stress

The qRT-PCR analysis showed that the expression level of *RcNAC72* was higher in the leaf, root and stem, but at its lowest level in the petal (Figure 1C). Analysis of the expression of *RcNAC72* under ABA treatment indicated that it was significantly and rapidly increased by 6–7-fold at 2 h, and by 12-fold at 24 h (Figure 1E), while drought, salt and low temperature treatments all slowly induced the expression of *RcNAC72* (Figure 1D,F,G). It is worth nothing that the expression of *RcNAC72* was not significantly induced until 24 h under drought and salt treatment, and it reached 14-fold compared with the control under drought treatment (Figure 1D,F). Similarly, the expression of *RcNAC72* gradually increased with time under low temperature treatment, reaching a 30–35-fold increase after 12–24 h (Figure 1G).

### 2.3. Promoter Analysis of RcNAC72

**Figure 2:**
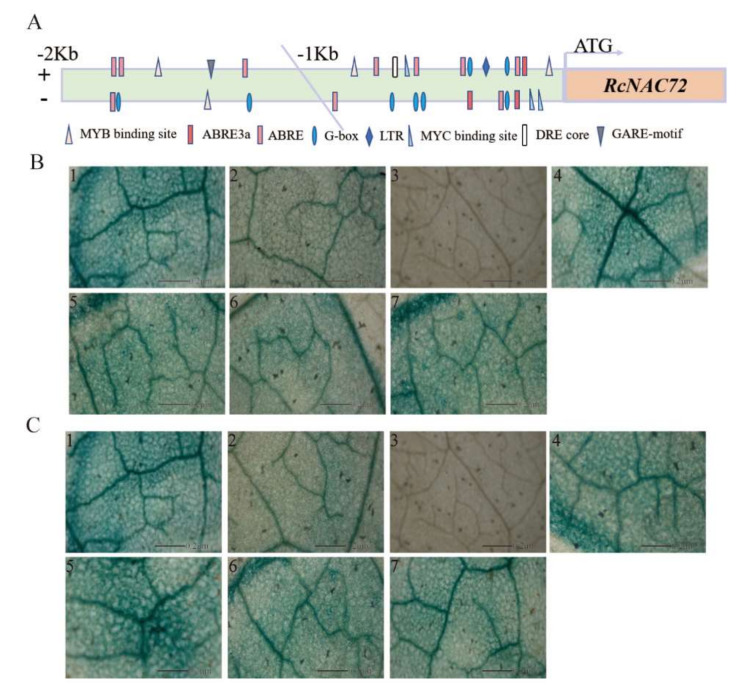
Analysis of promoter of *RcNAC72*. (**A**) Schematic diagram of the cis-acting elements of *RcNAC72* promoter. (**B**) Activity analysis of ProRcNAC72−1. (**1**) Positive control (The promoter of *CaMV35S* drove GUS gene expression). (**2**) The promoter of ProRcNAC72−1 drove GUS gene expression. (**3**) Negative control. (**4**) ABA treatment. (**5**) Drought treatment. (**6**) NaCl treatment. (**7**) Low temperature treatment. (**C**) Activity analysis of ProRcNAC72−2. (**1**) Positive control (The promoter of *CaMV35S* drove GUS gene expression). (**2**) The promoter of ProRcNAC72−2 drove GUS gene expression. (**3**) Negative control. (**4**) ABA treatment. (**5**) Drought treatment. (**6**) NaCl treatment. (**7**) Low temperature treatment. Scale bars, 0.2 μm.

The promoter sequence of *RcNAC72* 2000 bp upstream of the ATG start codon was cloned according to the genomic information on NCBI. There were various putative stress response and hormone response elements in the promoter region of *RcNAC72*, including ABRE (abscisic acid-response element), MYB (MYB binding site involved in drought inducibility), MYC (MYC binding site), LTR (low-temperature responsive element), DRE core (dehydration responsive element), G-box (light responsive element) and GARE-motif (gibberellin responsive element) (Figure 2A). These results indicate that the *RcNAC72* promoter may respond to abiotic stress.

The cloned promoter sequence was divided into two parts, each being a 1000 bp segment, using them to analyze the promoter activity. The results of the analysis of *RcNAC72* promoter activity in transiently transformed tobacco leaves demonstrate that tobacco leaves injected with ProNAC72-1 and ProNAC72-2 were a lighter blue than the positive control (Figure 2B(2),C(2),B(1),C(1)), and the negative control had no GUS gene expression (Figure 2B(3),C(3)). These results indicate that the two promoters of *RcNAC72* all have promoter activity and can drive GUS gene expression, but the intensity of driving GUS expression was lower than that of the positive control. The results of the effects of different stress treatments on the activity of the *RcNAC72* promoter show that the blue of tobacco leaves after stress treatment was consistently darker than that of unstressed leaves (Figure 2B(4)–(7),C(4)–(7)), indicating that the *RcNAC72* promoter responded to ABA, drought, salt and low temperature stress.

### 2.4. Silencing RcNAC72 by VIGS Reduced Dehydration Tolerance in Rose Leaf

**Figure 3:**
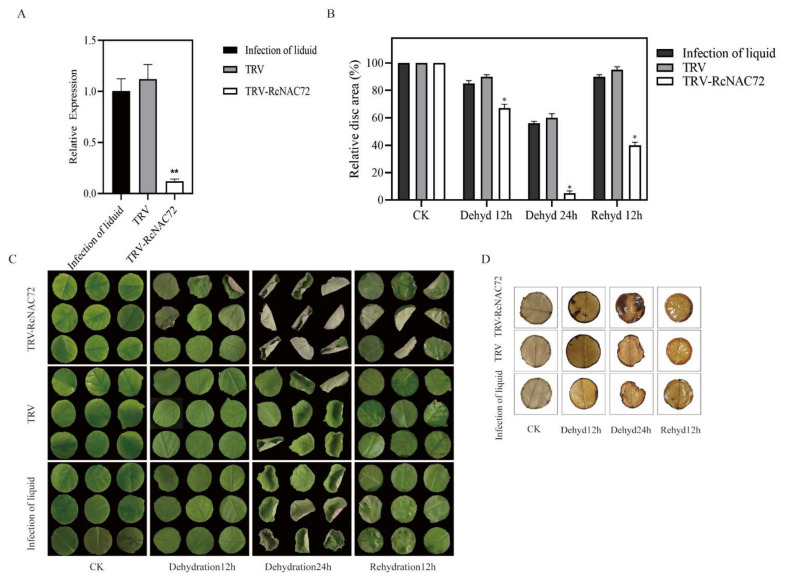
Silencing of *RcNAC72* in rose leaf discs by VIGS. (**A**). Detection of *RcNAC72* expression by qRT-PCR. (**B**). Relative areas of discs. (**C**). Phenotype of *RcNAC72*-silenced in rose leaf discs. (**D**). DAB staining. Three biological replications were performed. The bars show the SD. Asterisks indicate a significant difference ** *p* < 0.01 and * *p* < 0.5 compared with the corresponding controls.

By silencing *RcNAC72*, the role of *RcNAC72* in rose leaves was explored. The results show that compared with TRV and infection control, the expression of *RcNAC72* in the leaves was significantly reduced (Figure 3A), and the *RcNAC72* gene was successfully silenced. By dehydrating and rehydrating the *RcNAC72*-silenced rose discs, the results show that 85–90% of the leaves of the infection solution and TRV control were curled after 12 h of dehydration, while the *RcNAC72* silent discs were 67% curled, and there were significant differences (Figure 3B,C). The silent discs of *RcNAC72* were basically all curled, and the 60% discs of the infection solution and TRV control curled after 24 h of dehydration (Figure 3B,C). More than 90% of the infection solution and TRV control discs recovered completely, while the silent *RcNAC72* discs only recovered 40% after 12 h of rehydration (Figure 3B,C). DAB staining results demonstrate that *RcNAC72* silent discs were darker brown and had more O^2−^ production and H_2_O_2_ content (Figure 3D).

### 2.5. Overexpression of RcNAC72 in Arabidopsis Enhance Tolerance to Drought Stress

**Figure 4:**
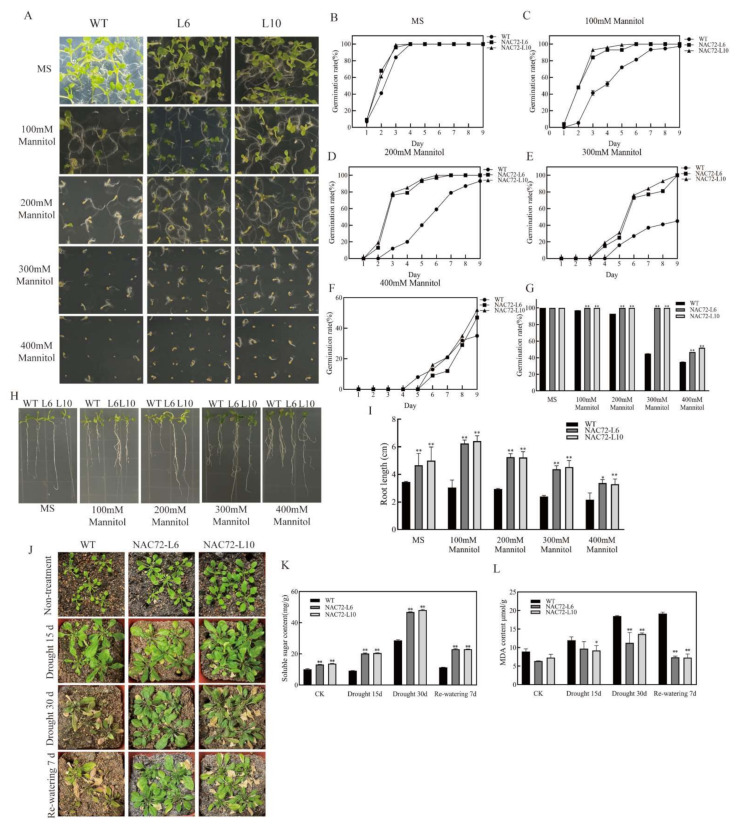
Seed germination, root length and phenotypes of WT and overexpression *RcNAC72* lines under drought treatment. (**A**). Seed germination of WT and overexpression *RcNAC72* lines with different concentrations of mannitol. (**B**). Germination rate in MS medium. (**C**). Germination rate in MS medium with 100 mM mannitol. (**D**). Germination rate in MS medium with 200 mM mannitol. (**E**). Germination rate in MS medium with 300 mM mannitol. (**F**). Germination rate in MS medium with 400 mM mannitol. (**G**). Statistics of germination rate under mannitol treatment. (**H**). Seed root length of WT and overexpression *RcNAC72* lines with different concentrations of mannitol. (**I**). Root length statistics under mannitol treatment. (**J**). Performance of WT and *RcNAC72* transgenic lines after drought treatment. (**K**). Soluble sugar content in WT and *RcNAC72* transgenic lines after drought treatment. (**L**). MDA content in WT and *RcNAC72* transgenic lines after drought treatment. Three biological replications were performed. The bars show the SD. Asterisks indicate a significant difference ** *p* < 0.01 and * *p* < 0.5 compared with the corresponding controls.

Two T3 *RcNAC72* transgenic lines, L6 and L10, with relatively high *RcNAC72* expression were selected among the 10 transgenic lines for subsequent analysis (Figure S2). Seeds of wild type (WT) and overexpression of *RcNAC72* (L6 and L10) were sown on MS medium containing 0, 100, 200, 300 and 400 mM mannitol. All seeds of different lines were able germinate on day 4, and there was no significant difference in germination rate, but seeds of L6 and L10 germinated faster in MS medium (Figure 4A,B,G). In MS medium supplemented with 100 and 200 mM mannitol, seed germination was slightly inhibited in both WT lines (97.33% and 93%), and all seeds of L6 and L10 germinated (Figure 4A,C,D). Seed germination of the WT line was significantly lower than that of the L6 and L10 strains in MS medium supplemented with both 300 and 400 mM mannitol. It is noteworthy that under 300 mM mannitol treatment, the germination rate of L6 and L10 was 100% on the ninth day, while the WT line was only 45% (Figure 4A,E–G). Similar results were found in the root length, which was significantly longer in both L6 and L10 under different drought treatments than in WT (Figure 4H,I). These above results indicate that *RcNAC72* transgenic plants could enhance the tolerance to drought to some extent.

Similarly, the drought stress tolerance of transgenic Arabidopsis was studied. For 30 day of drought, WT plants showed drought stress damage and partial death, while transgenic plants all showed green leaves and normal growth (Figure 4J). In addition, transgenic plants had higher soluble sugar content and lower MDA content than WT plants during drought stress (Figure 4K). In conclusion, we hypothesized that *RcNAC72* transgenic plants have enhanced tolerance to drought stress to some extent.

### 2.6. Overexpression of RcNAC72 in Arabidopsis Enhanced Sensitivity to ABA

**Figure 5:**
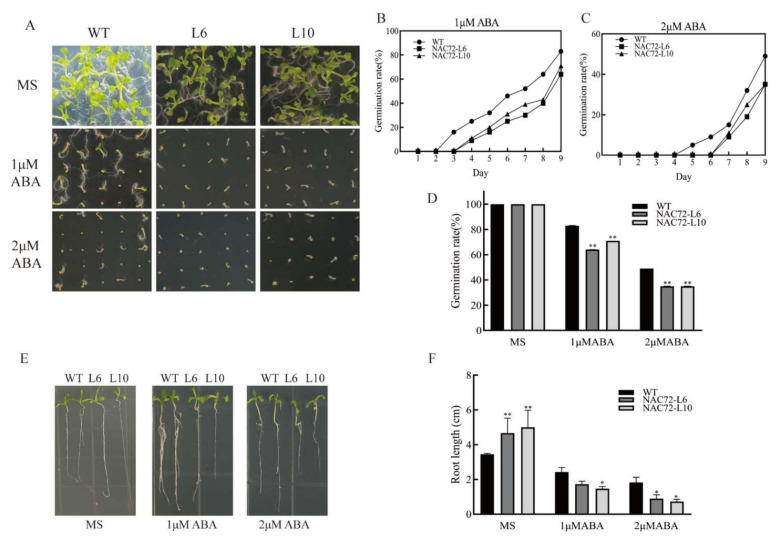
Seed germination and root length of WT and overexpression *RcNAC72* lines under ABA treatment. (**A**). Seed germination of WT and overexpression *RcNAC72* lines with different concentrations of ABA. (**B**). Germination rate in MS medium. (**C**). Germination rate in MS medium with 1 μM ABA. (**D**). Germination rate in MS medium with 2 μM ABA. (**E**). Seed root length of WT and overexpression *RcNAC72* lines with different concentrations of ABA. (**F**). Root length statistics under ABA treatment. Three biological replications were performed. The bars show the SD. Asterisks indicate a significant difference ** *p* < 0.01 and * *p* < 0.5 compared with the corresponding controls.

Based on the above study, we hypothesized that *RcNAC72* may be involved in the ABA pathway, and the sensitivity of *RcNAC72* transgenic plants to ABA was investigated. Seeds of WT and two *RcNAC72* transgenic lines were sown on MS plates supplemented with 0, 1 and 2 μM ABA. In MS plates, there was no significant difference except that the two transgenic lines germinated somewhat faster than WT. With the increase in added ABA concentration, the germination rate was inhibited in all (Figure 5A,D). On MS plates supplemented with 1 μM ABA, the germination rate of WT was 83% after 9 days, compared to 64% and 71% for the two transgenic lines (Figure 5B). When the concentration of ABA in MS plates was 2 μM, the germination rate of WT was only half after 9 days, while that of the two transgenic lines was only 35% (Figure 5C). Similar results were found in the root experiment. After the addition of ABA, the root length of two transgenic lines was significantly shorter than WT (Figure 5E,F). Therefore, we suggested that *RcNAC72* transgenic plants are more sensitive to ABA than WT plants.

### 2.7. Altered Expression of Stress-Related Genes in Overexpression RcNAC72 Arabidopsis Plants

**Figure 6:**
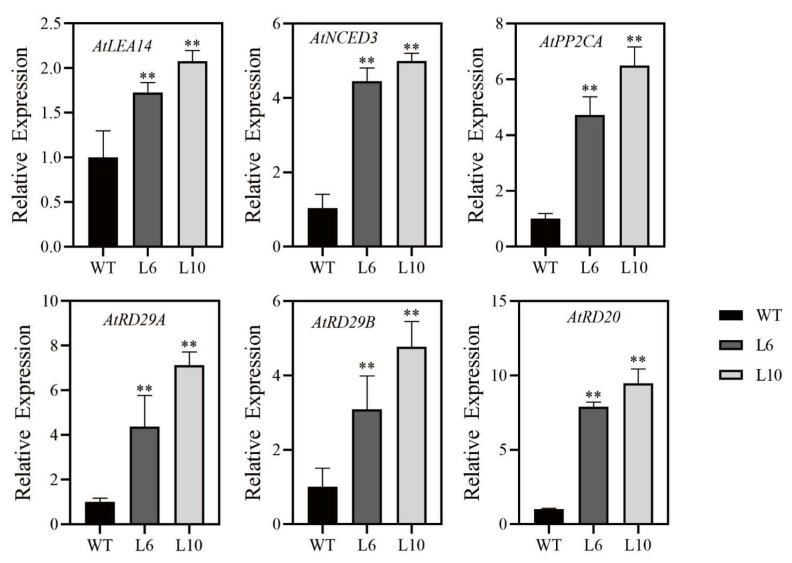
Expression levels of the stress-related genes in WT and *RcNAC72* transgenic plants under normal condition. Three biological replications were performed. The bars show the standard deviation (SD). Asterisks indicate a significant difference ** *p* < 0.01 compared with the corresponding controls.

The expression of *RcNAC72* induced by PEG, ABA, NaCl, and cold, shown in Figure 1, and *RcNAC72* transgenic Arabidopsis plants enhanced tolerance to drought and increased ABA sensitivity. We further assessed six stress-responsive genes (*AtLEA14*, *AtNCED3*, *AtPP2CA*, *AtRD29A*, *AtRD29B*, *AtRD20*) in the transgenic plants to determine the function of *RcNAC72* in the stress response. The qRT-PCR results indicate higher gene expression levels in *RcNAC72* transgenic plants than WT plants (Figure 6). We suppose that the transcription of these genes could be affected by *RcNAC72*.

### 2.8. RcABF4 Combines with the Promoter Region of RcNAC72 and RcNAC72 Interacts with RcDREB2A

**Figure 7:**
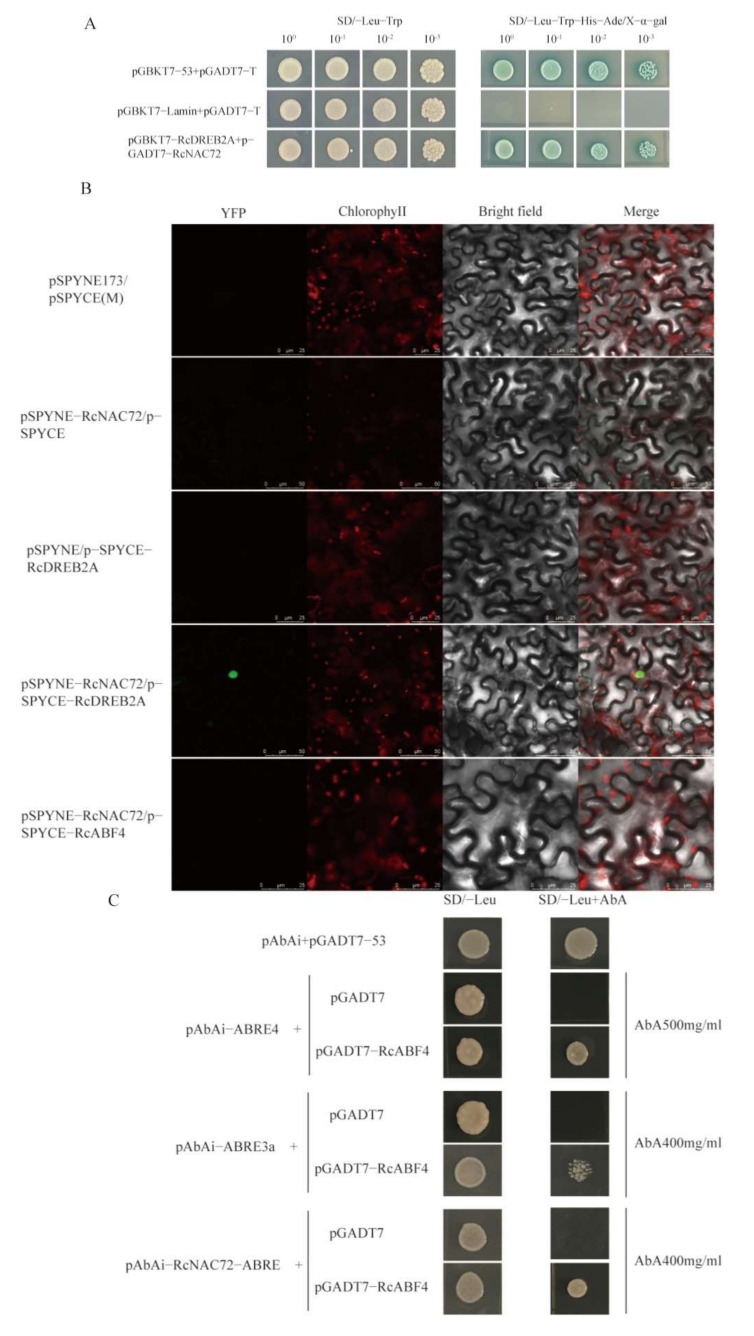
Verification that *RcDREB2A* and *RcABF4* have a regulatory relationship with *RcNAC72*. (**A**). Y2H assay of *RcNAC72* and *RcDREB2A*. (**B**). BiFC analysis of *RcNAC72* and *RcDREB2A*. The scale bar is 25 and 50 μm, respectively. (**C**). Y1H analysis of *RcABF4* binding to *RcNAC72* promoter.

The sequence of *RcNAC72* is significantly similar to that of Arabidopsis *ANAC72*. According to the STRING database, genes that interact with *ANAC72* are predicted (Figure S3A). Additionally, based on the correlation between rose drought transcriptome expression, it is speculated that *RcDREB2A* and *RcABF4* may interact with *RcNAC72* (Figure S3B). Yeast two-hybrid assays revealed that yeast transformed with pGBKT7-*RcDREB2A*+pGADT7-*RcNAC72* grew well on the selection medium SD/-Leu-Trp-His-Ade-x-α-gal and appear blue (Figure 7A), demonstrating that *RcNAC72* can interact with *RcDREB2A* in yeast. The yeast cells with pGBKT7-53 and pGADT7-T were positive controls, and those with pGBKT7-Lamin and pGADT7-T were negative controls. Since *RcABF4* was a transcriptional activator, 30 mM 3-AT cannot inhibit it (Figure S3C,D), and yeast two-hybrid assays could not be performed. The bimolecular fluorescence complementation (BiFC) assay revealed that the YFP signals are observed in the nuclei of tobacco leaves co-expressing *RcNAC72* and *RcDREB2A*, while no YFP signals are detected in negative control pSPYNE173/pSPYCE(M), pSPYNE-*RcNAC72*/pSPYCE, pSPYNE173/pSPYCE-*RcDREB2A* and pSPYNE-*RcNAC72*/pSPYCE-*RcABF4* (Figure 7B). These results confirm that *RcNAC72* could interact with *RcDREB2A*, but *RcNAC72* could not interact with *RcABF4* in vivo.

Promoter region of *RcNAC72* contained multiple ABRE cis-acting elements. Studies have shown that the promoter region containing ABRE cis-acting basically participated in the ABA pathway [37]. Combined with the correlation analysis of transcriptome expression, we speculated that *RcNAC72* may have a regulatory relationship with *RcABF4*. ABRE, ABRE3a, ABRE4 cis-acting elements and partial promoter fragments containing ABRE were inserted into the pAbAi vector. It was found that the minimal inhibitory concentration of Aureobasidin A (AbA) for bait yeast strains was 400, 500 and 400 mg mL^−1^, respectively. Since the ABRE element could not be inhibited by AbA, it could not be used for Y1H (Figure S4). Yeast cells transformed with pGADT7-*RcABF4*/pAbAi-ABRE3a, pGADT7-*RcABF4*/pAbAi-ABRE4 and pGADT7-*RcABF4*/pAbAi-*RcNAC72*-ABRE grew well on SD/-Leu with a corresponding concentration of AbA (Figure 7C). These results demonstrate that *RcABF4* could recognize ABRE cis-acting elements and bind the promoter of *RcNAC72*.

## 3. Discussion

In the present study, a drought-responsive NAC transcription factor *RcNAC72* was identified, which was a homolog of Arabidopsis *ANAC072*. Consistent with the reported NAC transcription factors, *RcNAC72* contained a conserved *N-*terminal and a *C*-terminal with a transcriptional activation activity domain [38]. Subcellular localization elucidated that *RcNAC72* is a nuclear localization protein, as with most transcription factors. *RcNAC72* was confirmed to respond to drought, salt and low temperature stresses, as well as to exogenous ABA. Analysis of the promoter of *RcNAC72* revealed that the promoter region contains multiple ABRE-like cis-acting elements. This result may explain why *RcNAC72* can rapidly respond to exogenous ABA. The promoter of *RcNAC72* has promoter activity and responded to abiotic stresses. The results of studies on a variety of plants indicate that stress-inducible promoters can provide stronger stress tolerance than the *CaMV35S* [39]. Promoter of *RcNAC72* can next be substituted for the *CaMV35S* stable transfer Arabidopsis for stress treatment of T3 generation. It can be further verified that the promoter of *RcNAC72* can be a candidate stress-inducible promoter for enhancing stress tolerance in plants.

Arabidopsis transgenic to the *RcNAC72* gene exhibited higher germination rates and longer root lengths than wild-type Arabidopsis under drought treatment. On the other hand, transgenic Arabidopsis had higher levels of sugars and MDA content, which play essential roles in plant resistance to drought stress. Furthermore, *RcNAC72* transgenic plants had higher expression of stress response genes, suggesting that the *RcNAC72* gene may enhance stress tolerance by regulation downstream stress response genes. It was shown that the promoter region of the selected stress response genes contained NAC recognition sites [27]. This result of transgenic Arabidopsis show that overexpression of *RcNAC72* enhanced the tolerance of Arabidopsis to drought stress. This was consistent with the results of most *NACs* functional studies [19,20,40]. In addition, leaf discs silencing *RcNAC72* gene were less able to recover after water loss stress and contained more oxygen accumulation. This result reinforces that the *RcNAC72* gene plays an active role in the resistance to drought stress.

In previous studies, most *NACs* were responsive to exogenous ABA and confirmed that *NACs* are involved in the ABA signaling pathway [21,27,41]. Similarly, *RcNAC72* responded rapidly to exogenous ABA, and transgenic Arabidopsis had lower germination rates and shorter toot lengths on the ABA-added medium, indicating that transgenic Arabidopsis was more sensitive to ABA. Moreover, Y1H assay confirmed that *RcABF4* can bind to the ABRE cis-acting element in the *RcNAC72* promoter region. Taken together, these results suggest that *RcNAC72* is involved in the ABA signaling pathway in response to drought stress. In Arabidopsis, *ANAC096* intercropped with *AtABF2* and *AtABF4* [14], ANAC019 and *ANAC055* were bound either *AtABF3* or *AtABF4* [42]. *ANAC072* cooperated *AtABF3* to regulate ABA-responsive gene regulation [33]. The results of this study are consistent with results of previous works. Apart from that, it has been shown that *NACs* interact with DREB transcription factors [34,43,44]. The *RcNAC72*-*RcDREB2A* interactions in rose were demonstrated through experiments. Therefore, we speculated that *RcNAC72* is involved in the DREB/CBF-COR pathway in addition to the ABA signaling pathway in response to drought stress in rose. Meanwhile, the promoter of *RcNAC72* contains a DRE cis-acting element, and whether *RcDREB2A* will recognize the *RcNAC72* promoter needs further verification, because *LlDREB1* can recognize the promoter of *LlNAC2* in lilies [35].

**Figure 8:**
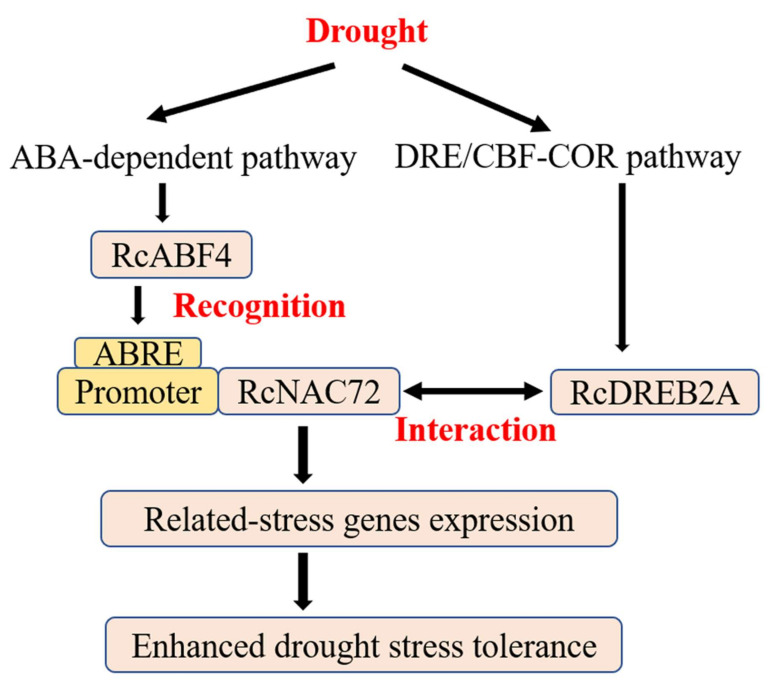
Schematic representation of *RcNAC72* expression in response to drought stress in roses. *RcABF4* recognizes the promoter of *RcNAC72*, and *RcNAC72* interacts with *RcDREB2A*, under drought stress. *RcNAC72* is involved in the ABA-dependent pathway and the DRE/CBF-COR pathway.

In summary, *RcNAC72* can respond to a variety of abiotic stresses and can enhance drought resistance and sensitivity to ABA in transgenic Arabidopsis, and silencing *RcNAC72* in rose leaves resulted in reduced leaf expansion capacity. *RcABF4* specifically recognizes the promoter of *RcNAC72*, and *RcNAC72* interacts with *RcDREB2A*, implying that *RcNAC72* is involved in both the ABA signaling pathway and the DREB/CBF-COR pathway. *RcNAC72* participates in the synergistic pathway to assist plants in responding to environmental stresses quickly and effectively. The regulatory network of *RcNAC72* in response to drought stress in roses is summarized in Figure 8. In Arabidopsis, *AtDREB2A* was involved in both ABA-independent and ABA-dependent pathway [45]. Further elucidation on whether *RcDREB2A* is involved in the ABA-dependent pathway can follow in future research.

In conclusion, we verified that silencing *RcNAC72* in rose leaves reduces the tolerance to water loss stress and rehydration. In addition, overexpression of *RcNAC72* Arabidopsis enhanced drought tolerance and sensitivity to ABA. We elucidated the regulatory mechanism of *RcNAC72* through ABA-dependent signaling pathway and the DRE/CBF-COR pathway in response to drought stress. That is, *RcABF4* specifically recognized the promoter of *RcNAC72*, while *RcNAC72* interacted with *RcDREB2A* in response to drought stress.

## 4. Materials and Methods

### 4.1. Plant Materials

The preparation method of *R. chinenesis* ‘Old Blush’ materials used in this study was described in our previous study [36]. Normally growing rose leaves, petals, roots and stems were removed and quickly putted into liquid nitrogen and stored at −80 °C for tissue specific analysis of *RcNAC72*. These seedlings were placed in a cooler at 4 °C as a low temperature treatment. Then, 20%PEG, 100 μM NaCl, and 100 μM ABA solutions were poured onto these seedlings as drought, salt, and ABA treatments. The leaves of these seedlings were taken at 0, 2, 4, 8, 12 and 24 h after treatment for the expression analysis of *RcNAC72* in response to abiotic stress. Three biological replications were performed. The arabidopsis (*Arabidopsis thaliana* Columnia-0) and tobacco (*Nicotiana benthamiana*) preparation and planting methods were in line with Yong [35].

### 4.2. Cloning and Sequencing Analysis of RcNAC72

Based on the previous drought transcriptome data and the genome data on NCBI, through ORF Finder (http://www.ncbi.nlm.nih.gov/gorf/gorf.html accessed on 20 December 2021) and BLAST (http://blast.ncbi.nlm.nih.gov accessed on 20 December 2021) to determine the full length of *RcNAC72*. Specific primers were designed to amplify the full length of *RcNAC72* (Table S1). The PCR amplified product was recovered by cutting the gel and connected with Zero Background pTOPO-Blunt Cloning Kit (Aidlab Biotech, Beijing, China). The sequenced plasmids were used as templates for subsequent experiments.

Amino acid multiple sequence alignments were performed by DNAMAN (version 7, LynnonBiosoft, San Ramon, CA, USA). Phylogenetic tree was constructed via MEGA5 using neighbor-joining method. ProtParam (http://web.expasy.prg/protparam/ accessed on 20 December 2021) was used to predict protein molecular weight and isoelectric point.

### 4.3. RNA Extraction and Quantitative Real-Time PCR

RNA of *R. chinensis* under each treatment was extracted by liquid nitrogen grinding using the Easy Spin Plus RNA Extraction Kit (RN53, Aidlab, Beijing, China). The reverse transcription kit PC54-TRUEscript RT kit (+gDNA Eraser) (Aidlab, Beijing, China) was applied to reverse transcription of RNA into cDNA. Primer Premier 5.0 was employed to design fluorescent quantitative PCR primers, and the primers were shown in Table S1. TAKARA’s TB Green^®^ Premix Ex TaqTM II (Takara, Shiga, Japan) and Bio-Rad/CFX Connect TM Real-Time Detection System (Bio-Rad, Hercules, CA, USA) were used to qRT-PCR detection, referring to the instructions for reaction system. The relative expression level adopted the 2^−^^△△Ct^ method, and *RcPP2A* was the internal reference gene.

Leaves of WT and *RcNAC72* transgenic and Arabidopsis under normal conditions were used to detect the expression of relevant stress genes by qRT-PCR detection. Three biological replications were performed.

### 4.4. RcNAC72 Promoter Cloning, Cis-Acting Elements and Promoter Activity Analysis

According to the genome information on NCBI, Primer 5.0 was used to design specific primers to amplify the sequence of 2000 bp upstream of “ATG”. Plant CARE (http://bioinfonnatics.psb.ugent.be/webtools/plantcare/html/ accessed on 20 December 2021) was used to predict the cis-acting elements of the promoter sequence.

The cloned 2000 bp *RcNAC72* promoter was divided in to two parts, and the 1000 bp parts were named ProNAC72-1 and ProNAC72-2, respectively. The ProNAC72-1 and ProNAC72-2 promoter sequence were inserted separately between *Sca*I and *Bam*HI of the pBI121-CaMV35S-GUS vector to construct the promoter expression vector pBI121-ProNAC72-1-GUS and pBI121-ProNAC72-2-GUS, by using ClonExpressII One Step Cloning Kits (Vazyme, Piscataway, NJ, United States). The pBI121-ProNAC72-1-GUS, pBI121-ProNAC72-2-GUS and pBI121-CaMV35S-GUS vectors were transformed into Agrobacterium tumefaciens GV3101 and the infection solutions were prepared respectively and injected into the tobacco leaves. The related primers are shown in Table S1. After the dark culture for one day, the tobacco leaves after injection were treated by spraying with clean water, ABA (100 μM), mannitol (100 mM), NaCl (200 mM) and 4 °C. Tobacco leaves injected with an infestation solution that did not contain Agrobacteria, while the leaves were sprayed with distilled water as a negative control. After 24 h of treatment, the injected tobacco leaves were cut out, incubated with GUS stain at 37 °C for 12–24 h, then decolorized with 95% alcohol, observed and photographed with a stereo microscope.

### 4.5. Silencing of RcNAC72 in Rose Leave Discs by Virus-Induced Gene Silencing (VIGS)

The 415 bp sequence of the 3′UTR region of *RcNAC72* was inserted between *Eco*RI and *Bam*HI sites of the pTRV2 vector to construct the pTRV2-*RcNAC72* vector. The related primers are shown in Table S1. The specific procedure referred to the previous research [26]. The mature leaves of *R. chinensis* cutting seedlings were made into discs with a diameter of one centimeter using a hole punch. Discs were completely immersed in the infection solution and infiltrated under vacuum of 0.5 MPa for 20 min. The infection solution containing pTRV1 and pTRV2 (*v*:*v* = 1:1) was a negative control (TRV), and the infection solution without a vector was used to exclude the damage caused by the infection solution to the disc. Processing of discs after vacuuming was as described in the previous study [26]. Then, discs were dehydrated for 12 h, 24 h and rehydrated for 24 h. Before treatment, discs were sampled for qRT-PCR to test the silencing efficiency of VIGS.

The 3,30-diaminobenzidine (DAB) staining were performed according to the method described by Chen et al. [46]. Nine discs were used in each treatment, with three replicates, and the experiment was replicated three times.

### 4.6. Obtainment of Transgenic Arabidopsis with RcNAC72 and Functional Verification

The full length of *RcNAC72* without the stop codon was cloned into the pBI121-GFP vector with the *CaMV35S* promoter. The related primers are shown in Table S1. The pBI121-*RcNAC72*-GFP vector was transformed into Arabidopsis thaliana Columbia-0 (WT) plants according to the floral dip method [47]. Transgenic positive lines were screened on MS medium containing kanamycin (50 mg mL^−1^). Two homozygous lines of OE-6 and OE-10 with relatively high expression levels in the T3 generation were selected for gene function analysis.

In order to detect the germination rate of Arabidopsis thaliana under stress treatments, seeds of different lines of Arabidopsis were sown on MS medium supplemented with mannitol (0, 100, 200, 300 or 400 mM) and ABA (1, 2 or 3 μM). The germination rate was counted for 9 consecutive days. Each of the above treatments was carried out simultaneously, with three biological replicates for each treatment.

For determining drought tolerance and ABA sensitivity in root growth of transgenic plants, seeds grown in MS for 7 days and then transferred to MS medium containing mannitol (0, 100, 200, 300 or 400 mM) and ABA (1, 2 or 3 μM) [27]. The root length of each treatment was counted and photographed.

The 3-week-old seedlings were subjected to drought treatment. After being fully watered, the drought lasted for 30 days, and then the plants were re-watered for 7 days.

### 4.7. Subcellular Localization of RcNAC72

The full length of the *RcNAC72* gene with the terminator removed was inserted between the *Xho*I and *Sal*I sites of the pBI121-GFP vector, using ClonExpress II One Step Cloning Kits (Vazyme, Nanjing, China). The specific operations were in accordance with the instructions. The constructed vector pBI121-*RcDREB2A*-GFP and pBI121-GFP plasmids were transformed into Agrobacterium tumefaciens GV3101 and the infection solutions were prepared respectively and injected into the tobacco leaves. The injected tobacco leaves were cut into approximately 1 cm × 1 cm sizes, placed on a glass slide with 100μL ddH_2_O dripped in advance and covered with a cover glass. These leaves were imaged using a Leica TCS SP8 Confocal Laser Scanning Platform (Leica SP8, Leica, Buffalo Grove, IL, USA) under 488 mm laser excitation and 500–530 nm filter to observe the GFP positioning. The primers used above are listed in Table S1.

### 4.8. Transcription Activation Activity Analysis and Yeast Two-Hybrid Assay

According to the instructions of ClonExpressII One Step Cloning Kits (Vazyme, Nanjing, China), the full length, *N-*terminal (1–526 bp) and *C-*terminal (527–1059 bp) of the *RcNAC72* gene were inserted into the *Eco*RI and *Bam*HI of the pGBKT7 vector. The recombinant plasmids and pGADT7 plasmid were transferred into Y2HGold yeast cells (Huayueyang, Beijing, China), referring to the Quick Easy Yeast Transformation Mix kit instructions (Clontech, San Jose, CA, USA). The transformed yeast cells were diluted 10-fold with sterile water and 10 μL of the diluted solution was spotted on SD/-Trp-Leu and SD/-Trp-Leu-His-Ade-x-α-gal media, respectively. These were cultured upside down at 30 °C for 3 days, and the yeast growth was observed.

The full length of *RcDREB2A* was inserted into pGBKT7 vector as a prey, and the full length of *RcNAC72* was inserted into pGADT7 as a bait. As mentioned above, pGBKT7- *RcDREB2A* and pGADT7- *RcNAC72* plasmids were jointly transferred into Y2H yeast. These yeast cells were observed on SD/-Trp-Leu and SD/-Trp-Leu-His-Ade-x-α-gal selective media. The primers used above are listed in Table S1.

### 4.9. Bimolecular Fluorescent Complimentary (BiFC) Assay

Full-length *RcNAC72* was cloned into the pSPYNE173 vector, while *RcDREB2A* and *RcABF4* were cloned into the pSPYCE (M) vector. As described in the above subcellular localization test method, pSPYNE173-*RcNAC72* and pSPYCE-*RcDREB2A* were co-injected into tobacco leaves, along with pSPYNE173-*RcNAC72* and pSPYCE-*RcABF4*. The GFP signals were observed through Leica TCS SP8 Confocal Laser Scanning Platform (Leica SP8, Leica, USA). The primers used above are listed in Table S1.

### 4.10. Yeast One-Hybrid Assay

Y1H was completed by using the Matchmaker Gold Yeast One-Hybrid System Kit (TaKaRa, Beijing, China). Three tandem copies of ABRE (ACGTG), ABRE3a (TACGTG) and ABRE4 (CACGTA) were generated by oligonucleotide synthesis and inserted into the pAbAi bait vector. Similarly, the 455 bp sequence of *RcNAC72* promoter containing the ABRE element was cloned into the pAbAi bait vector. The CDS region of *RcABF4* was cloned into the pGADT7 prey vector to generate the pGADT7-*RcABF4* plasmid. The bait plasmids were linearized and co-transformed with the prey plasmid into the Y1HGold yeast. The interacting ones grew normally on the selective SD/-Leu + Aureobasidin A (AbA) medium. The primers used above are listed Table S1.

### 4.11. Physiological Parameters Measurements

The MDA content was measured following the method described previously [36]. The soluble sugar content was evaluated with related detection kits.

## Data Availability

All data supporting the findings of this study are available within the paper and within its supplementary materials published online.

## Supplementary Materials

The following supporting information can be downloaded at: https://www.mdpi.com/article/10.3390/ijms23031755/s1.

## References

[1] Farooq M., Wahid A., Kobayashi N., Fujita D., Basra S.M.A. (2009). Plant drought stress: Effects, mechanisms and management. Agron. Sustain. Dev..

[2] Razi K., Muneer S. (2021). Drought stress-induced physiological mechanisms, signaling pathways and molecular response of chloroplasts in common vegetable crops. Crit. Rev. Biotechnol..

[3] Thirumalaikumar V.P., Devkar V., Mehterov N., Ali S., Ozgur R., Turkan I., Mueller-Roeber B., Balazadeh S. (2018). NAC transcription factor JUNGBRUNNEN1 enhances drought tolerance in tomato. Plant Biotechnol. J..

[4] Singh D., Laxmi A. (2015). Transcriptional regulation of drought response: A tortuous network of transcriptional factors. Front. Plant Sci..

[5] Piao W., Sakuraba Y., Paek N.-C. (2019). Transgenic expression of rice MYB102 (OsMYB102) delays leaf senescence and decreases abiotic stress tolerance in Arabidopsis thaliana. BMB Rep..

[6] Cui M., Zhang W.J., Zhang Q., Xu Z.Q., Zhu Z.G., Duan F.P., Wu R. (2011). Induced over-expression of the transcription factor OsDREB2A improves drought tolerance in rice. Plant Physiol. Biochem..

[7] Garcia M.N.M., Cortelezzi J.I., Fumagalli M., Capiati D.A. (2018). Expression of the Arabidopsis ABF4 gene in potato increases tuber yield, improves tuber quality and enhances salt and drought tolerance. Plant Mol. Biol..

[8] Hong Y.B., Zhang H.J., Huang L., Li D.Y., Song F.M. (2016). Overexpression of a Stress-Responsive NAC Transcription Factor Gene ONACO22 Improves Drought and Salt Tolerance in Rice. Front. Plant Sci..

[9] Olsen A.N., Ernst H.A., Leggio L.L., Skriver K. (2005). NAC transcription factors: Structurally distinct, functionally diverse. Trends Plant Sci..

[10] Wang Z.Y., Dane F. (2013). NAC (NAM/ATAF/CUC) transcription factors in different stresses and their signaling pathway. Acta Physiol. Plant..

[11] An J.P., Yao J.F., Xu R.R., You C.X., Wang X.F., Hao Y.J. (2018). An apple NAC transcription factor enhances salt stress tolerance by modulating the ethylene response. Physiol. Plant..

[12] Sakuraba Y., Kim Y.-S., Han S.-H., Lee B.-D., Paek N.-C. (2015). The Arabidopsis Transcription Factor NAC016 Promotes Drought Stress Responses by Repressing AREB1 Transcription through a Trifurcate Feed-Forward Regulatory Loop Involving NAP. Plant Cell.

[13] Tran L.S.P., Nakashima K., Sakuma Y., Simpson S.D., Fujita Y., Maruyama K., Fujita M., Seki M., Shinozaki K., Yamaguchi-Shinozaki K. (2004). Isolation and functional analysis of Arabidopsis stress-inducible NAC transcription factors that bind to a drought-responsive cis-element in the early responsive to dehydration stress 1 promoter. Plant Cell.

[14] Xu Z.-Y., Kim S.Y., Hyeon D.Y., Kim D.H., Dong T., Park Y., Jin J.B., Joo S.-H., Kim S.-K., Hong J.C. (2013). The Arabidopsis NAC Transcription Factor ANAC096 Cooperates with bZIP-Type Transcription Factors in Dehydration and Osmotic Stress Responses. Plant Cell.

[15] Wang Q., Guo C., Li Z.Y., Sun J.H., Deng Z.C., Wen L.C., Li X.X., Guo Y.F. (2021). Potato NAC Transcription Factor StNAC053 Enhances Salt and Drought Tolerance in Transgenic Arabidopsis. Int. J. Mol. Sci..

[16] Mergby D., Hanin M., Saidi M.N. (2021). The durum wheat NAC transcription factor TtNAC2A enhances drought stress tolerance in Arabidopsis. Environ. Exp. Bot..

[17] Ma J., Wang L.Y., Dai J.X., Wang Y., Lin D. (2021). The NAC-type transcription factor CaNAC46 regulates the salt and drought tolerance of transgenic Arabidopsis thaliana. BMC Plant Biol..

[18] Yuan X., Wang H., Cai J.T., Bi Y., Li D.Y., Song F.M. (2019). Rice NAC transcription factor ONAC066 functions as a positive regulator of drought and oxidative stress response. BMC Plant Biol..

[19] Guan H., Liu X., Niu F., Zhao Q., Fan N., Cao D., Meng D., He W., Guo B., Wei Y. (2019). OoNAC72, a NAC-Type Oxytropis ochrocephala Transcription Factor, Conferring Enhanced Drought and Salt Stress Tolerance in Arabidopsis. Front. Plant Sci..

[20] Huang Q.J., Wang Y., Li B., Chang J.L., Chen M.J., Li K.X., Yang G.X., He G.Y. (2015). TaNAC29, a NAC transcription factor from wheat, enhances salt and drought tolerance in transgenic Arabidopsis. BMC Plant Biol..

[21] Chen J., Gong Y., Gao Y., Zhou Y.B., Chen M., Xu Z.S., Guo C.H., Ma Y.Z. (2021). TaNAC48 positively regulates drought tolerance and ABA responses in wheat (*Triticum aestivum* L.). Crop J..

[22] Wang L., Hu Z., Zhu M., Zhu Z., Hu J., Qanmber G., Chen G. (2017). The abiotic stress-responsive NAC transcription factor SlNAC11 is involved in drought and salt response in tomato (*Solanum lycopersicum* L.). Plant Cell Tissue Organ Cult..

[23] Jian W., Zheng Y.X., Yu T.T., Cao H.H., Chen Y., Cui Q.Y., Xu C., Li Z.G. (2021). SlNAC6, A NAC transcription factor, is involved in drought stress response and reproductive process in tomato. J. Plant Physiol..

[24] Li M., Wu Z.Y., Gu H., Cheng D.W., Guo X.Z., Li L., Shi C.Y., Xu G.Y., Gu S.C., Abid M. (2021). AvNAC030, a NAC Domain Transcription Factor, Enhances Salt Stress Tolerance in Kiwifruit. Int. J. Mol. Sci..

[25] Dai F., Zhang C., Jiang X., Kang M., Yin X., Lu P., Zhang X., Zheng Y., Gao J. (2012). RhNAC2 and RhEXPA4 Are Involved in the Regulation of Dehydration Tolerance during the Expansion of Rose Petals. Plant Physiol..

[26] Jiang X., Zhang C., Lu P., Jiang G., Liu X., Dai F., Gao J. (2014). RhNAC3, a stress- associated NAC transcription factor, has a role in dehydration tolerance through regulating osmotic stress- related genes in rose petals. Plant Biotechnol. J..

[27] Ding A., Li S., Li W., Hao Q., Wan X., Wang K., Liu Q., Liu Q., Jiang X. (2019). RhNAC31, a novel rose NAC transcription factor, enhances tolerance to multiple abiotic stresses in Arabidopsis. Acta Physiol. Plant..

[28] Zhao Q., Zhong M., He L., Wang B., Liu Q.L., Pan Y.Z., Jiang B.B., Zhang L. (2018). Overexpression of a chrysanthemum transcription factor gene DgNAC1 improves drought tolerance in chrysanthemum. Plant Cell Tissue Organ Cult..

[29] Yamaguchi-Shinozaki K., Shinozaki K. (2006). Transcriptional regulatory networks in cellular responses and tolerance to dehydration and cold stresses. Annu. Rev. Plant Biol..

[30] Zhang X., Long Y., Chen X.X., Zhang B.L., Xin Y.F., Li L.Y., Cao S.L., Liu F.H., Wang Z.G., Huang H. (2021). A NAC transcription factor OsNAC3 positively regulates ABA response and salt tolerance in rice. BMC Plant Biol..

[31] Luo P., Chen Y., Rong K., Lu Y., Wang N., Xu Z., Pang B., Zhou D., Weng J., Li M. (2021). ZmSNAC13, a maize NAC transcription factor conferring enhanced resistance to multiple abiotic stresses in transgenic Arabidopsis. Plant Physiol. Biochem. PPB.

[32] Liu W., Zhao B.-G., Chao Q., Wang B., Zhang Q., Zhang C., Li S., Jin F., Yang D., Li X. (2019). Function analysis of ZmNAC33, a positive regulator in drought stress response in Arabidopsis. Plant Physiol. Biochem..

[33] Li X.Y., Li X.L., Li M.J., Yan Y.C., Liu X., Li L. (2016). Dual Function of NAC072 in ABF3-Mediated ABA-Responsive Gene Regulation in Arabidopsis. Front. Plant Sci..

[34] Yu M., Liu J., Du B., Zhang M., Wang A., Zhang L. (2021). NAC Transcription Factor PwNAC11 Activates ERD1 by Interaction with ABF3 and DREB2A to Enhance Drought Tolerance in Transgenic Arabidopsis. Int. J. Mol. Sci..

[35] Yong Y., Zhang Y., Lyu Y. (2019). A Stress-Responsive NAC Transcription Factor from Tiger Lily (LlNAC2) Interacts with LlDREB1 and LlZHFD4 and Enhances Various Abiotic Stress Tolerance in Arabidopsis. Int. J. Mol. Sci..

[36] Jia X., Feng H., Bu Y., Ji N., Lyu Y., Zhao S. (2021). Comparative Transcriptome and Weighted Gene Co-expression Network Analysis Identify Key Transcription Factors of Rosa chinensis ʻOld Blushʼ After Exposure to a Gradual Drought Stress Followed by Recovery. Front. Genet..

[37] Yoshida T., Mogami J., Yamaguchi-Shinozaki K. (2014). ABA-dependent and ABA-independent signaling in response to osmotic stress in plants. Curr. Opin. Plant Biol..

[38] Nuruzzaman M., Sharoni A.M., Kikuchi S. (2013). Roles of NAC transcription factors in the regulation of biotic and abiotic stress responses in plants. Front. Microbiol..

[39] Hou L., Chen L.J., Wang J.Y., Xu D.F., Dai L.X., Zhang H., Zhao Y.X. (2012). Construction of Stress Responsive Synthetic Promoters and Analysis of Their Activity in Transgenic Arabidopsis thaliana. Plant Mol. Biol. Rep..

[40] Wu H., Fu B., Sun P., Xiao C., Liu J.-H. (2016). A NAC Transcription Factor Represses Putrescine Biosynthesis and Affects Drought Tolerance. Plant Physiol..

[41] Fujita M., Fujita Y., Maruyama K., Seki M., Hiratsu K., Ohme-Takagi M., Tran L.S.P., Yamaguchi-Shinozaki K., Shinozaki K. (2004). A dehydration-induced NAC protein, RD26, is involved in a novel ABA-dependent stress-signaling pathway. Plant J..

[42] Hickman R., Hill C., Penfold C.A., Breeze E., Bowden L., Moore J.D., Zhang P.J., Jackson A., Cooke E., Bewicke-Copley F. (2013). A local regulatory network around three NAC transcription factors in stress responses and senescence in Arabidopsis leaves. Plant J..

[43] Shaheen T., Mahmood ur R., Abbas M.F., Zaib P., Mehboob ur R., Ullah I., ul Qamar M.T., Arif A. (2018). Identification, Characterization, Homology Modeling and Protein-Protein Interactions of Cotton (*Gossypium arboreum*) DREB Gene. Int. J. Agric. Biol..

[44] Jin C., Li K.Q., Xu X.Y., Zhang H.P., Chen H.X., Chen Y.H., Hao J., Wang Y., Huang X.S., Zhang S.L. (2017). A Novel NAC Transcription Factor, PbeNAC1, of Pyrus betulifolia Confers Cold and Drought Tolerance via Interacting with PbeDREBs and Activating the Expression of Stress-Responsive Genes. Front. Plant Sci..

[45] Kim J.S., Mizoi J., Yoshida T., Fujita Y., Nakajima J., Ohori T., Todaka D., Nakashima K., Hirayama T., Shinozaki K. (2011). An ABRE Promoter Sequence is Involved in Osmotic Stress-Responsive Expression of the DREB2A Gene, Which Encodes a Transcription Factor Regulating Drought-Inducible Genes in Arabidopsis. Plant Cell Physiol..

[46] Chen K., Song M., Guo Y., Liu L., Xue H., Dai H., Zhang Z. (2019). MdMYB46 could enhance salt and osmotic stress tolerance in apple by directly activating stress-responsive signals. Plant Biotechnol. J..

[47] Clough S.J., Bent A.F. (1998). Floral dip: A simplified method for Agrobacterium-mediated transformation of Arabidopsis thaliana. Plant J. Cell Mol. Biol..

