# Supplementary material for: Drought-Responsive NAC Transcription Factor RcNAC72 Is Recognized by RcABF4, Interacts with RcDREB2A to Enhance Drought Tolerance in Arabidopsis
Source: Int J Mol Sci. 2022 Feb 3;23(3):1755. doi: 10.3390/ijms23031755 (PMC8915184; doi:10.3390/ijms23031755)
Supplement: Supplementary file 1 [file ijms-23-01755-s001.zip › ijms-1566516-supplementary.pdf]

Supplementary Figure S1-S4:

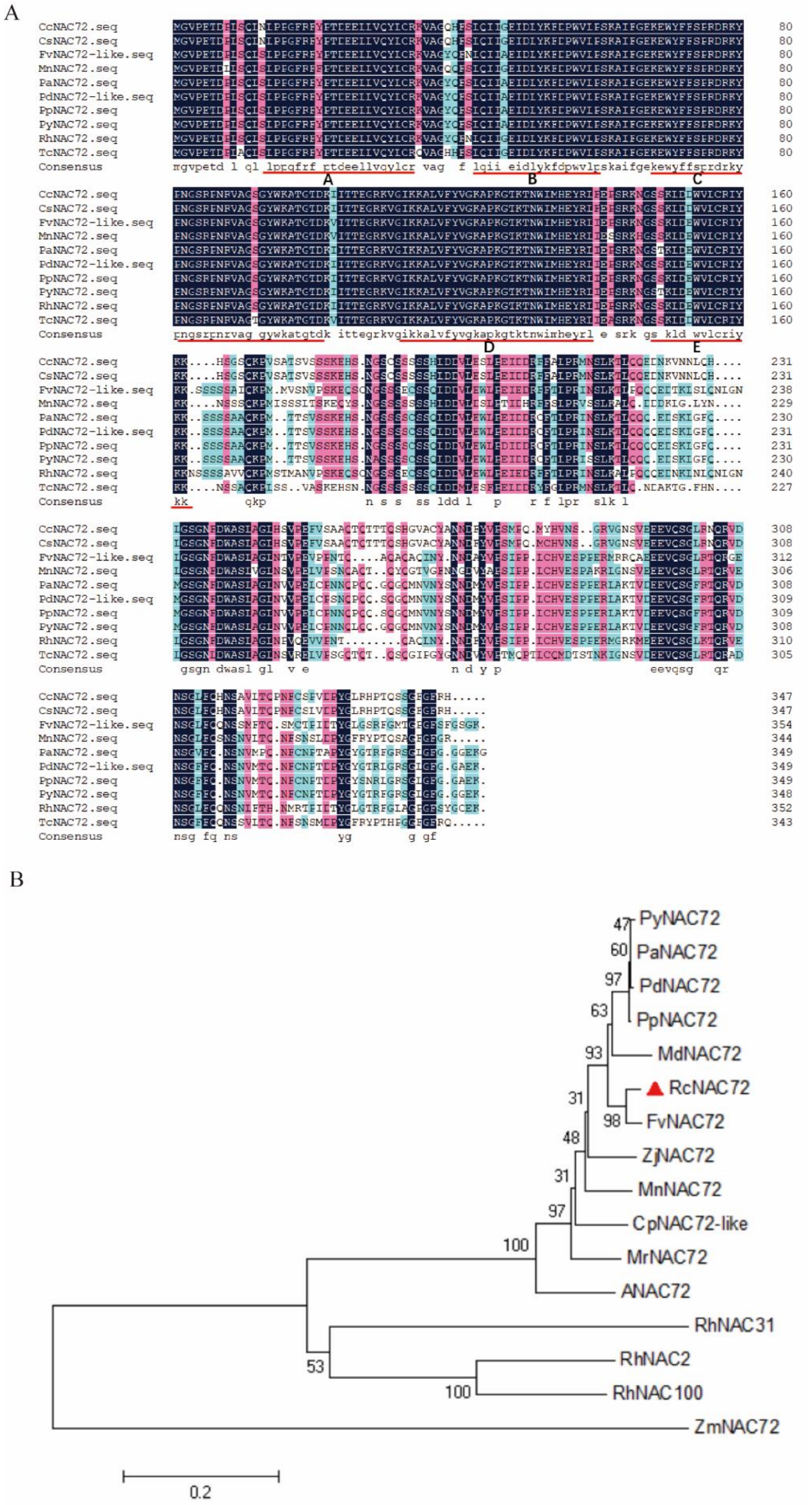

Figure S1. Multiple sequence alignments and phylogenetic analysis of *RcNAC72*. (A) Multiple sequence alignment of *RcNAC72*. Red lines indicate the locations of the five highly conserved subdomains A-E. (B) Phylogenetic tree analysis of *RcNAC72*. Protein

sequences used in multiple sequence alignments and phylogenic tree analysis are shown in Supplementary Table S2.

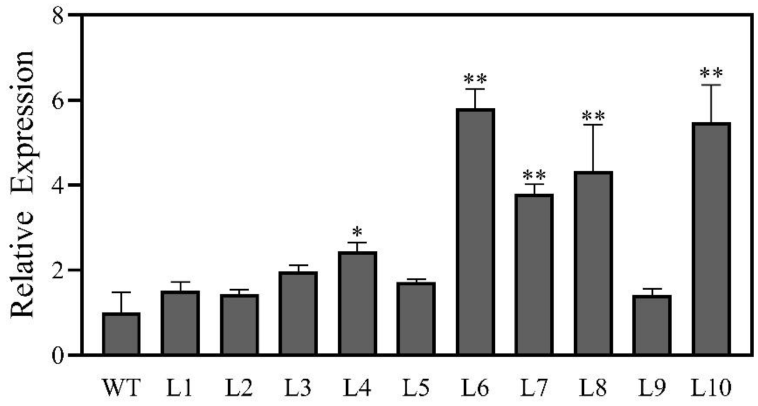

Figure S2. qRT-PCR screening of *RcNAC72* transgenic Arabidopsis. Three biological replications were performed. The bars show the standard deviation (SD). Asterisks indicate a significant difference **p<0.01 and *<0.5 compared with the corresponding controls.

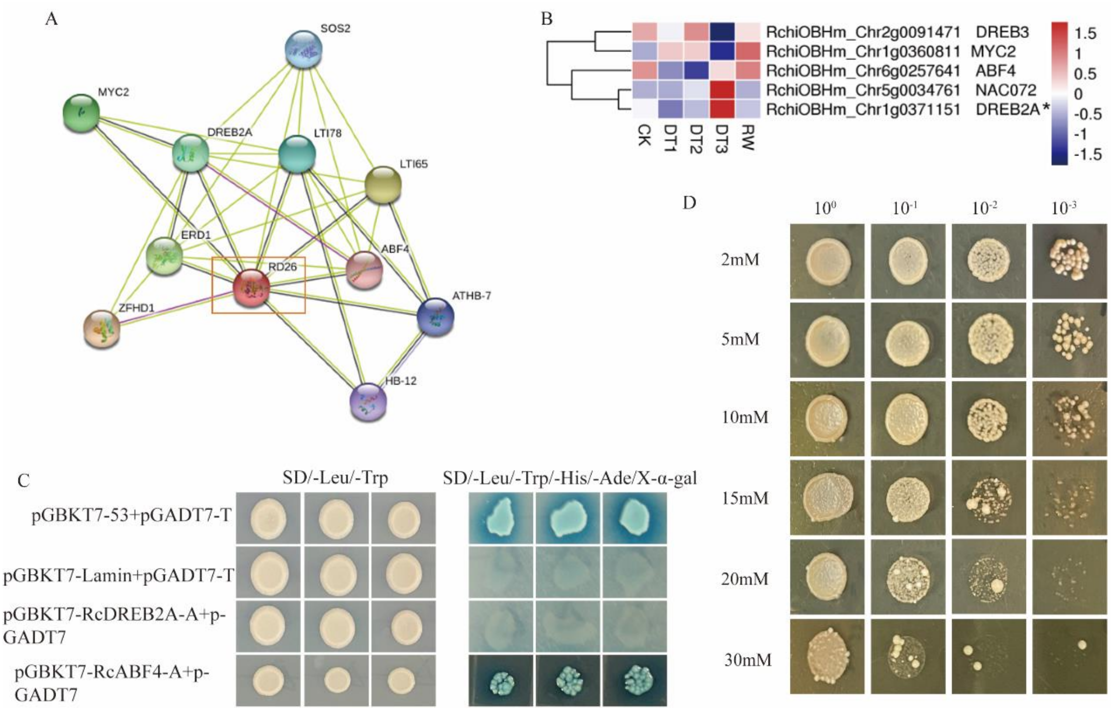

Figure S3. Screening for genes with possible regulatory relationship with *RcNAC72*. (A). Network diagram of the presence of regulatory genes in ANAC72 of Arabidopsis. (B). Heat map analysis of drought stress transcriptome expression of genes that may have a regulatory relationship with RcNAC72. (C). Transcriptional activation of *RcDREB2A* and *RcABF4*. (D). Screening for 3-AT concentrations that inhibit the transcriptional activation activity of *RcABF4*.

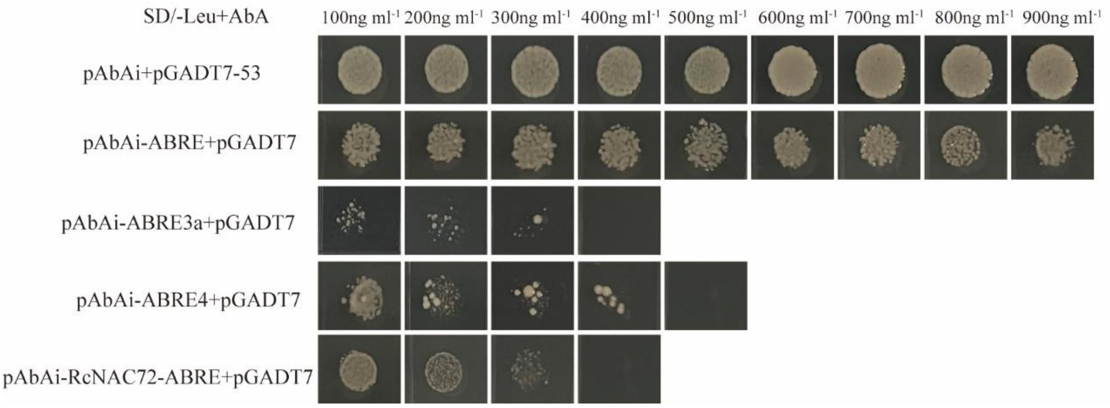

Figure S4. Test of bait yeast strain for AbAr expression.

Supplementary Table S1-S2:

Table S1 Related primers

Name of primer	Primer sequence (5’-3’)
RcNAC72-F	ATGGGCGTGCCGGAAACCGACCCT
RcNAC72-R	TCACTTTTCACAGCCGTAGC
RcNAC72-1-F	GAATCTCGGGTCGGGCA
RcNAC72-1-R	TGGGCTTGGGTATTGGGA
RcPP2A-F	GAGGACAGGACCAGGAAGG
RcPP2A-R	GCTCTACGCCGCCAACAT
ProNAC72-F	TTGATACAGACGGATTGCTACG
ProNAC72-R	TTCTTCTGGTCAGAGCTTGAG
pBI121-ProNAC72-1-GUS-F	TTTCTCAAGATCAGAAGTACTTTGACCACTAGCTTTTTACAATTCTCA
pBI121-ProNAC72-1-GUS-R	CATAAGGGACTGACCACCCGGGGATCCTTCTTCTGGTCAGAGCTTGAGATTT
pBI121-ProNAC72-2-GUS-F	TTTCTCAAGATCAGAAGTACTTTGATACAGACGGATTGCTACGTC
pBI121-ProNAC72-1-GUS-R	CATAAGGGACTGACCACCCGGGGATCCTTGTGTGTTAAAGAGGCTGGAGAC
pTRV2-RcNAC72-F	GTGAGTAAGGTTACCGAATTCCGGTAGGGCAAAAAAAAGGAG
pTRV2-RcNAC72-R	CGTGAGCTCGGTACCGGATCCTTATTGAACAATAAAAGATTGCTAAATTCT

pBI121-RcNAC72-GFP-F	CATTTACGAACGATACTCGAGATGGGCGTGCCGGAAACCGACC C
pBI121-RcNAC72-GFP-R	CACCATCACTAGTACGTCGACCTTTTCACAGCCGTAGC
pGBKT7-RcNAC72-A-F	CATGGAGGCCGAATTCATGGGCGTGCCGGAAAC
pGBKT7-RcNAC72-A-R	GCAGGTCGACGGATCCCTTTTCACAGCCGTAGCTGA
pGBKT7-RcNAC72-C-F	ATGGAGGCCGAATTCCGATGGCAAACGTTCCGA
pGBKT7-RcNAC72-C-R	GCAGGTCGACGGATCCCTTTTCACAGCCGTAGCTGA
pGBKT7-RcNAC72-N-F	CATGGAGGCCGAATTCATGGGCGTGCCGGAAAC
pGBKT7-RcNAC72-N-R	GCAGGTCGACGGATCCTCGACATCGGCTTCTGTACTACTG
pGBKT7-RcDREB2A-F	ATGGAGGCCGAATTCATGGGTGCTTATGATCAAGGTTC
pGBKT7-RcDREB2A-R	CCGCTGCAGGTCGACGGATCCGTTTGCATTGCCGCCGCA
pGADT7-RcNAC72-F	GTACCAGATTACGCTCATATGATGGGCGTGCCGGAAACC
pGADT7-RcNAC72-R	CAGCTCGAGCTCGATGGATCCCTTTTCACAGCCGTAGCTGAAA
pSPYNE173-RcNAC72-F	TGGCGCGCCACTAGTGGATCCATGGGCGTGCCGGAAACC
pSPYNE173-RcNAC72-R	TTGCTCCATCCCGGGAGCGGTACCCTTTTCACAGCCGTAGCTGA A
pSPYCE (M)-RcDREB2A-F	CCCAGGCCTACTAGTGGATCCATGGGTGCTTATGATCAAGGTTC
pSPYCE (M)-RcDREB2A-R	TTCGAGCTCCTACCCGGGAGCGGTACCGTTTGCATTGCCGCCGC A
pSPYCE (M)-RcABF4-F	AGGCCTACTAGTGGATCCATGGGATCTAATATAAACTTCAAGAA

pSPYCE (M)-RcABF4-R	GAGCTCCTACCCGGGAGCGGTACCCCAAGGGCCAGTCAATGTTC
pAbAi-ABRE-F	CACGTGACGTGACGTGC
pAbAi-ABRE-R	TCGAGCACGTCACGTCACGTGGTAC
pAbAi-ABRE3a-F	CTACGTGTACGTGTACGTGC
pAbAi-ABRE3a-R	TCGAGCACGTACACGTACACGTAGGTAC
pAbAi-ABRE4-R	CCACGTACACGTACACGTAC
pAbAi-ABRE4-F	TCGAGTACGTGTACGTGTACGTGGGTAC
pProRcNAC72ABRE-AbAi-F	CTTGAATTCGAGCTCGGTACCTCTCTCTACGTGCCGAATTGACC
pProRcNAC72ABRE-AbAi-R	ATACAGAGCACATGCCTCGAGGTGAAACGAACCCACGTCGG
AD-RcABF4-F	GTACCAGATTACGCTCATATGATGGGATCTAATATAAACTTCAAGAACTATG
AD-RcABF4-R	CAGCTCGAGCTCGATGGATCCCCAAGGGCCAGTCAATGTTC
AtActin-F	CTCATGCCATCCTCCGTCTT
AtActin-R	ACTTGCCCATCGGGTAATTC
AtRD29A -F	GAGCAACGAGGGGAAGATAAAAG
AtRD29A-R	TCAGTCGCACCACCACCGAACCA
AtRD29B-F	CAAAACCAAGCACCTACACA
AtRD29B-R	CTCCTTCACTCCACTTCCAC
AtRD20-F	ATTCGAGCACCTATGACACC
AtRD20-R	AAACTTCCATCAAAGCAACC

AtLEA14-F	ACCGGATTTAATTCATTAAGCGCT
AtLEA14-R	TCCCAAGCTGGCAGAGGGAAT
AtNCED3-F	GGATTGATGCTCCAGATTG
AtNCED3-R	CGGACGGCGAGTTGATT
AtPP2CA-F	AACGGTGTAGCCATTCC
AtPP2CA-R	ACATCCCATAGTCCATCA

Table S2 Accession numbers of genes used in this study

Gene name	Accession number
FvNAC72	XP_004291667.1
RhNAC2	AFQ21786.1
RhNAC100	AFS95065.1
RhNAC31	MF576436
PdNAC72	XP_034210680.1
PpNAC72	XP_020418173.1
PyNAC72	XP_034210680.1
PaNAC72	XP_021809122.1
MnNAC72	XP_010089503.1
MdNAC72	ADL36797.1
ZjNAC72	XP_031287408.1
MrNAC72	KAB1217220.1
CpNAC72-like	XP_021894680.1
AtNAC72	AT4G27410.2
ZmNAC72	KJ727003.1
AtRD29A	NM_124610
AtRD29B	NM_124609
AtRD20	NM_128898
AtLEA14	At1G01470
AtNCED3	NM_112304.3

AtPP2CA	NM_111974.4
AtActin	NM_112764
